# Supplementary figures and images for: NEMO- and RelA-dependent NF-κB signaling promotes small cell lung cancer
Source: Cell Death Differ. 2023 Jan 18;30(4):938–51. doi: 10.1038/s41418-023-01112-5 (PMC10070460; doi:10.1038/s41418-023-01112-5)

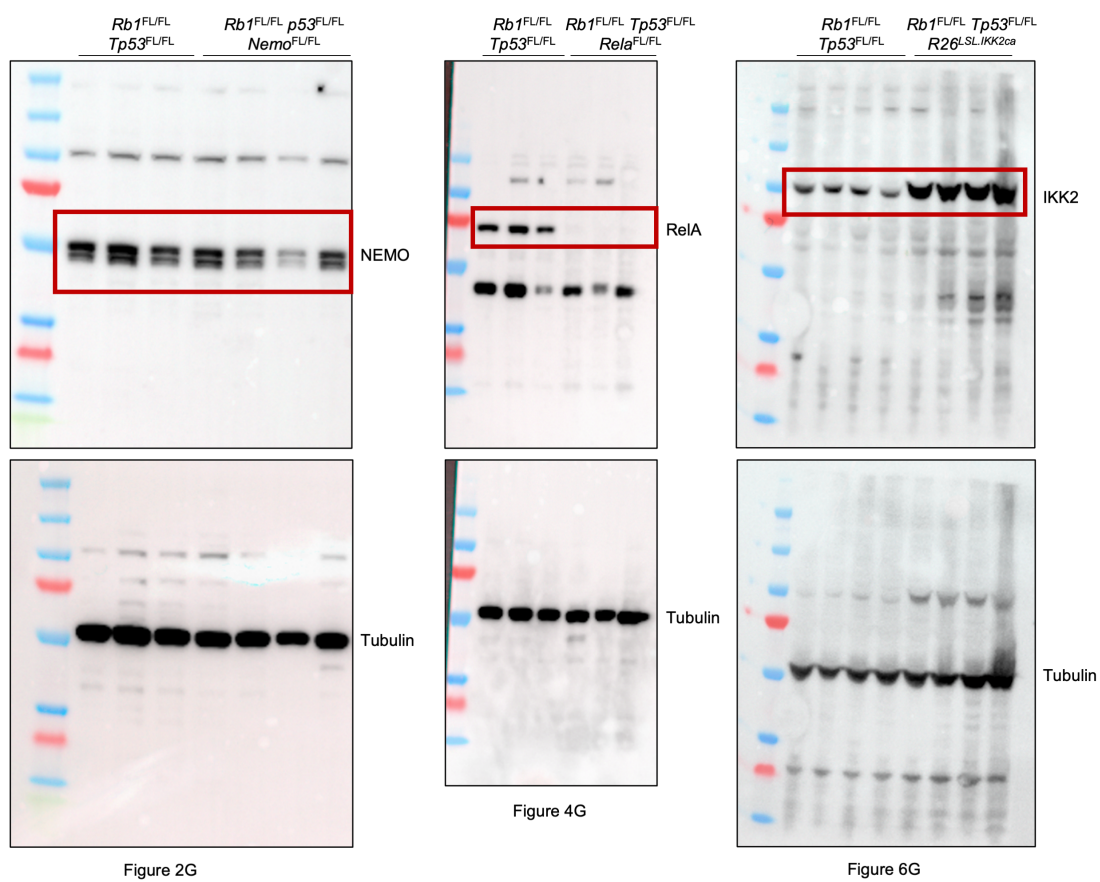

**Supplementary Figure 1.** Uncropped gels of all immunoblots presented in the manuscript.

Supplement: Supplementary file 2 — Supplementary Figure 1 [file 41418_2023_1112_MOESM2_ESM.pdf]
